# Exercises Based on a Non-Immersive Virtual Reality Application for Upper Limb Rehabilitation

**DOI:** 10.3390/bioengineering12070726

**Published:** 2025-07-01

**Authors:** Cosmin-Ilie Cotia, Silviu Dan Mandru

**Affiliations:** Faculty of Automotive, Mechatronics and Mechanical Engineering, Technical University of Cluj-Napoca, Bd. Muncii, Nr. 103–105, 400641 Cluj-Napoca, Romania; dan.mandru@mdm.utcluj.ro

**Keywords:** virtual reality, rehabilitaiton, 3D vision, unity, body traking, sensors, upper limb, exercise

## Abstract

Virtual reality (VR) technologies have gained increasing attention in the field of physical rehabilitation due to their potential to enhance patient engagement and provide adaptive, feedback-rich environments. In this study, we report on the development and preliminary evaluation of a VR-based rehabilitation application aimed at improving upper extremity function, including muscle strength, endurance, and joint mobility. The application delivers a structured set of interactive exercises designed to support recovery through engaging, gamified tasks with real-time performance feedback and scalable difficulty levels. A pilot usability study was conducted with a cohort of target users to assess the system’s practicality, therapeutic relevance, and user satisfaction. Qualitative data were collected to evaluate usability, effectiveness, and areas for further improvement. Preliminary results suggest that the VR application is usable, accessible, and well-received by users, with high levels of engagement reported throughout the intervention. Participants also provided constructive feedback, emphasizing the potential benefits of incorporating enhanced sensory feedback mechanisms to improve immersion and therapeutic impact. These initial findings support the viability of VR-based rehabilitation tools and provide a foundation for future clinical studies aimed at validating their efficacy in diverse patient populations.

## 1. Introduction

Non-immersive VR systems which do not rely on head-mounted displays have demonstrated promising results in upper limb rehabilitation, particularly for post-stroke patients. Evidence from recent clinical trials indicates that such systems can significantly enhance motor recovery and patient motivation when compared to conventional therapies [1]. These findings highlight the potential of non-immersive VR as an effective and accessible alternative, minimizing the cognitive and visual fatigue sometimes associated with fully immersive solutions. Systems incorporating VR offer diverse functionalities, including avatar-based feedback, real-time performance tracking, and tailored exercises for individual patients [2]. Studies have also demonstrated that VR is effective in alleviating anxiety and depression while enhancing patients’ overall emotional well-being [3], particularly when body involvement is integrated to enhance emotional effects [4]. Furthermore, VR-based therapeutic interventions have also been used for neurorehabilitation, such as in the treatment of schizophrenia and dementia, by providing cognitive stimulation in a controlled virtual settings [5]. Virtual reality (VR) technologies have been increasingly integrated into rehabilitation settings, offering immersive and non-immersive solutions for upper limb recovery. Recent systematic reviews emphasize the growing prevalence of immersive VR systems in stroke rehabilitation, typically leveraging Unity 3D and Oculus Quest platforms to create gamified, task-oriented exercises [6,7]. However, these solutions often require standalone VR headsets, higher costs, and technical expertise, which may limit their adoption in low-resource or clinical environments.

Non-immersive VR platforms have emerged as a viable alternative, particularly when paired with motion sensors to facilitate remote rehabilitation for conditions such as chronic low back pain [8]. These systems demonstrated improvements in pain, mobility, and adherence, supporting their potential use in broader physical therapy contexts. Furthermore, recent findings highlight that low-cost motion-capture systems using VR accessories (e.g., HTC Vive trackers) can yield joint kinematic measurements comparable in repeatability to those from high-end marker-based systems like Vicon, especially for functional movements such as squatting [9].

Despite these advancements, few studies have explored the integration of non-immersive VR systems specifically designed for upper limb rehabilitation post-stroke. Our study addresses this gap by introducing a cost-effective, non-immersive VR application for upper limb motor training. Unlike immersive systems, our approach prioritizes clinical accessibility and ease of integration without sacrificing the capability to deliver quantitative movement feedback. This work builds on current literature while providing a novel system architecture tailored to rehabilitation workflows with minimal setup and training overhead.

Clinical evidence supporting VR and AR in rehabilitation has robustly demonstrated that the integration of gamification with VR and AR technologies significantly improved psychological and functional outcomes for orthopedic patients [10]. This aligns with the growing trend of applying modern VR technologies to interpret and interact with medical models, offering both anatomical insights and functional guidance [5]. For instance, during painful procedures such as burn wound dressing, VR has been shown to significantly reduce patients’ perceived pain levels by creating a distraction effect. This success is attributed to VR’s capacity to evoke positive emotional responses and enhance the sense of presence within the virtual environment [10].

This paper focuses on the integration of VR in rehabilitation engineering, specifically for upper limb recovery. We will present a functional prototype that incorporates exercises for arm rehabilitation.

## 2. Concept Development and System Architecture

**Figure 1:**
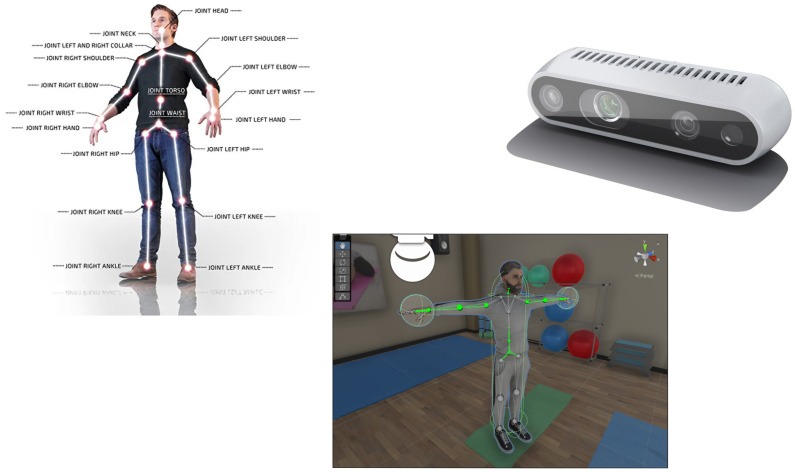
Body tracking system with a 3D vision camera.

The architecture of the system is shown in Figure 1. We chose to make a non-immersive prototype using a 3D vision camera. The Intel Realsense D435i 3D camera (Intel, California, CA, USA) was chosen, which combines distance measurement with inertial measurement, uses an IMU sensor (inertial measurement unit), and [11] has the possibility to measure transverse movements but also rotational movements with 6 degrees of freedom. Practical IMU sensors are a combination of several sensors with the capacity for gyroscopic measurement, which detect rotation, three-axis motion, inclination, or rotation around a point. The camera had an 87° × 58° field of view, making it possible to use it both indoors and outdoors. The ideal application distance is 0.3–3 [m], with an accuracy of 2% to 2 [m]. The illumination RGB had a resolution of 1920 × 1080, and the “frame rate” was 30 fps. Camera communication occurred on a USB - C * 3.1 Gen 1*. The 3D camera monitored the patient’s movement, and the movement information was processed using an SDK (software development kit) version v0.36.4 from NuiTrack [12].

### 2.1. Unity Components

The system was developed using Unity 2022.3, adopting a modular and real-time architecture. Key components integrated into the software architecture include the following:

- Unity XR Toolkit: Provided abstraction for hardware management and allowed seamless support for non-immersive VR deployment.
- Nuitrack SDK Integration: Enabled real-time skeletal tracking using the Intel RealSense D435i sensor, providing 3D joint positioning data for upper limb motion.
- Canvas UI System: Managed exercise selection, live feedback prompts, and navigation within the user interface.
- Animation Rigging and Inverse Kinematics (IK): Was applied to animate the avatar model, enabling real-time pose mirroring based on tracked user data.

### 2.2. Avatar-Based Motion Representation

The system utilizes a real-time animated avatar within the Unity environment to mirror the user’s skeletal posture and upper limb movements. The avatar model is fully rigged and responds to joint tracking data provided by the RealSense camera through the Nuitrack SDK. Using Unity’s animation rigging and inverse kinematics chains, the avatar emulates user movements with anatomical accuracy and responsiveness.

Feedback to the user is provided through two primary channels:

- Visual Feedback: When the user successfully completes a movement within the predefined angular threshold (±15° from the target), a “Target Reached” message is displayed alongside a green confirmation marker. This validation cue confirms correct execution and reinforces positive behavior. In cases of deviation, visual trajectory paths and posture indicators appear, helping guide realignment.
- Auditory Feedback: The system emits a positive audio cue to confirm a completed motion and a corrective sound if the movement deviates from expected trajectories. This dual-channel system enhances engagement and provides multimodal learning reinforcement.
  The avatar feedback system relies on a structured mapping of skeletal joint data obtained from the Intel RealSense D435i camera via the Nuitrack SDK. The SDK provides real-time 3D tracking of 26 upper-body joints, including the head, shoulders, elbows, wrists, and hands. These tracked coordinates are mapped onto a fully rigged humanoid avatar in Unity using the Transform Constraint and Animation Rigging components.
  Each joint from the real-world skeleton is linked to a corresponding bone in the avatar hierarchy—for example, the user’s right elbow is mapped to the RightForearm bone of the avatar. This direct correspondence ensures that the avatar accurately mirrors user movements in real time. To preserve anatomical plausibility, inverse kinematics (IK) chains are applied, allowing the avatar to maintain joint coherence and natural motion transitions.

### 2.3. Hardware and Software Architecture Diagrams and Flow

**Figure 2:**
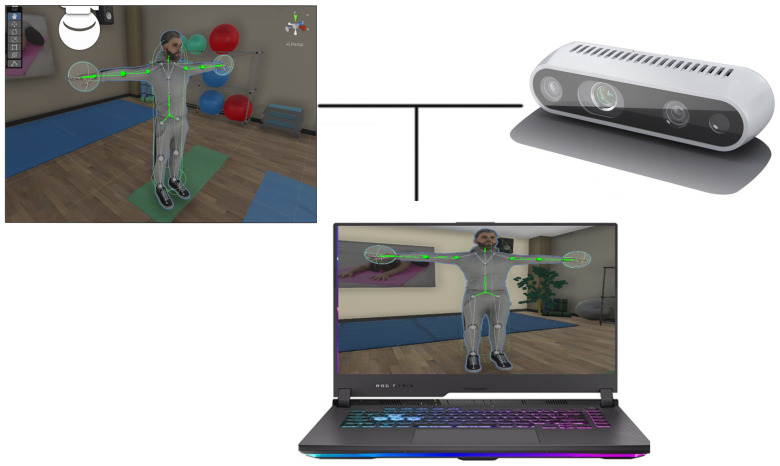
Hardware architecture of the system.

**Figure 3:**
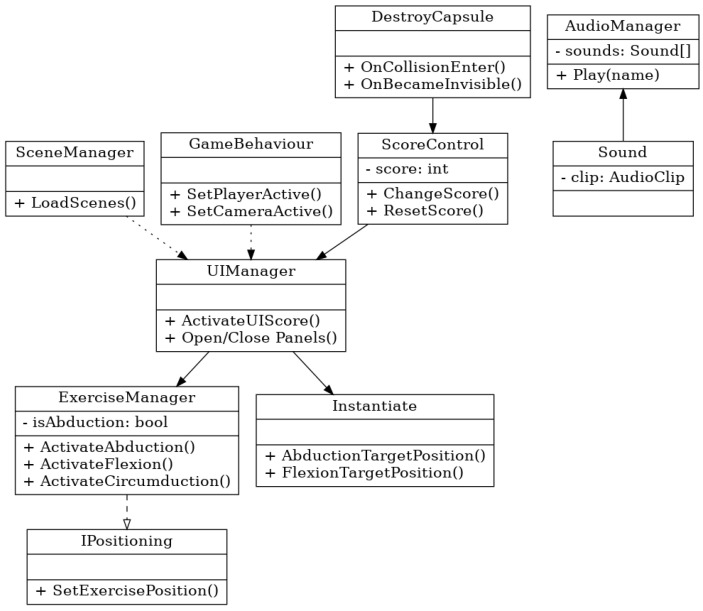
Software architecure and functional logic.

The system is built on a modular pipeline that integrates depth sensing hardware, middleware SDKs, and a Unity-based rehabilitation environment. The hardware architecture includes an Intel RealSense D435i depth camera that captures real-time three-dimensional skeletal data of the user. This sensor is capable of identifying 26 anatomical keypoints, including critical upper limb joints such as the shoulder, elbow, wrist, and hand. The camera feeds this information into a processing unit—in this case, a laptop running the Unity application—that serves as the central node of interaction, visualization, and computation. Figure 2 below illustrates the complete architecture of hardware components:

The internal architecture of the application is structured around a modular, event-driven model implemented in Unity using C#. Each class manages a specific functional layer in the rehabilitation workflow, with well-defined responsibilities and communication pathways. The system design follows principles of component decoupling and reuse, making it both maintainable and extensible. The full architecture is shown in Figure 3:

At the core of the control logic is the ExerciseManager, which acts as the central controller for exercise routines. This manager handles the initialization and activation of specific movement modules such as ActivateAbduction(), ActivateFlexion(), and ActivateCircumduction(). It interacts directly with the IPositioning interface to align the spatial logic of exercises within the virtual scene. Depending on the type of movement selected, target positions are computed and instantiated in real-time via the Instantiate module. For example, invoking AbductionTargetPosition() dynamically generates virtual targets for abduction-range tracking within the scene.

To track and respond to exercise performance, the ScoreControl class functions as a logical bridge between motion validation and system feedback. It continuously monitors whether the user’s joints reach or align with expected target coordinates. Upon successful alignment, the ChangeScore() method updates the internal score counter and triggers reward logic, including textual feedback (“Target Reached”) and updates to the on-screen score. The class also provides symmetry-based methods like ChangeScoreLeft() and ChangeScoreRight() to track bilateral performance when needed. The architecture is designed to be intuitive for end users while remaining flexible and scalable, supporting potential extension.

### 2.4. Tracking Setup and Environment Configuration

#### 2.4.1. Dephth Camera Framing and Selection Zone

**Figure 4:**
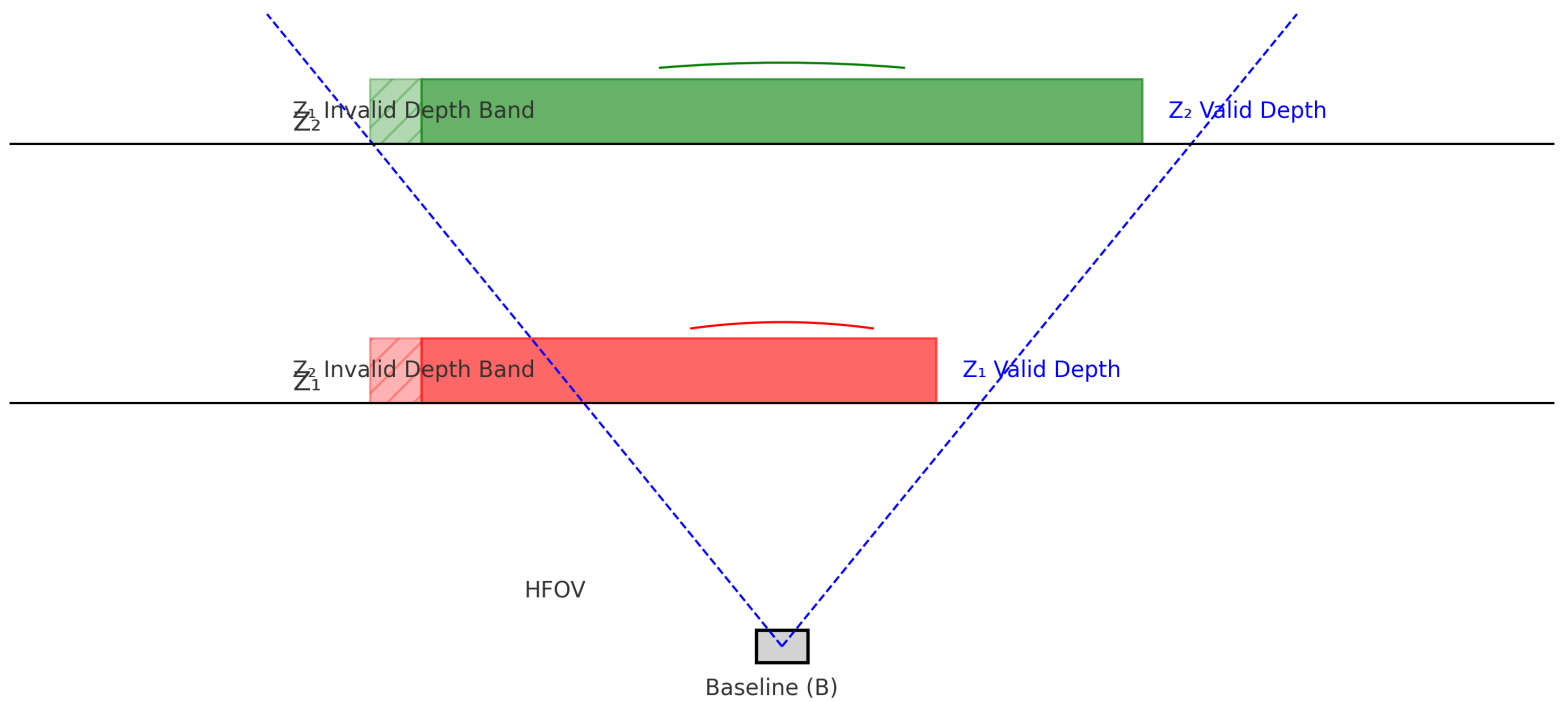
Framing objects in the field of view of the 3D camera.

Alternative input methods such as Leap Motion controllers have also been explored for remote motor rehabilitation of upper limbs, demonstrating effective motion capture and patient engagement [13]. The movements captured by the Intel RealSense D435i 3D camera are processed using the Nuitrack SDK [12], a middleware solution for skeletal tracking and gesture recognition. The avatar serves as a visual representation of the user’s movements and is integrated into a suite of interactive rehabilitation applications designed to encourage motor engagement and adherence to therapeutic exercises. As illustrated in Figure 4, the visual field of the 3D camera is carefully calibrated to optimize tracking accuracy. Objects and body segments detected within the green-highlighted area represent the ideal tracking zone typically within the recommended operational range of 0.3 to 3 m from the camera sensor. Maintaining the subject within this zone ensures optimal depth sensing, minimizes tracking errors, and allows the system to deliver reliable motion feedback. This framing is crucial for ensuring consistent data capture during rehabilitation sessions, particularly when evaluating complex upper-limb movements.

**Figure 5:**
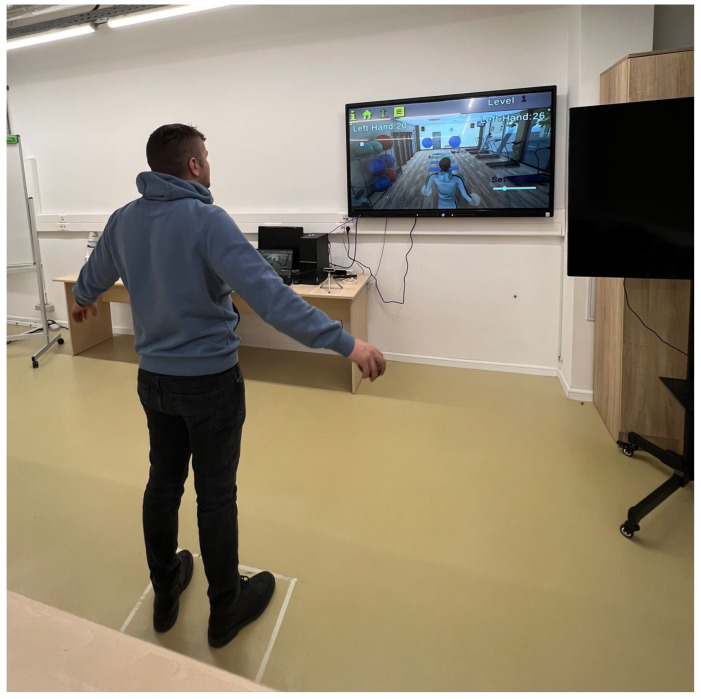
Camera selection zone.

It is recommended that the camera not be exposed directly to natural light in the field of view. It is also recommended that the person on whom the movement will be analyzed avoid dark clothing. The most common rehabilitation applications using augmented reality and virtual reality are game type or task type. In game-based approaches, patients are more attracted and interested in the recovery process, but also increase their well-being. In task-based applications, patients oftern experience a sense of responsability, which can contrubute to increased sefl-confidence. In this research, we have included applications such as play and the type of task being performed. Such applications often follow a gamified approach or involve task performance, both shown to improve motivation and well-being [14]. The hardware structure used consists of a processing unit, the Intel RealSense D435i camera, and an LED display where the activity of the movement generated by the patient will be projected, see Figure 5 below.

#### 2.4.2. User Interface and User Selection

**Figure 6:**
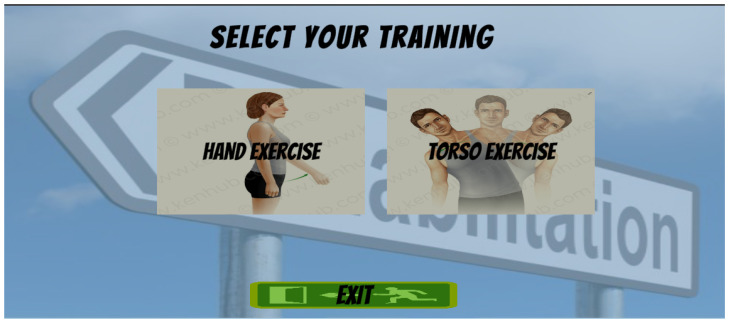
User Interface for training selection.

The application features a structured and intuitive graphical user interface (GUI) Figure 6 designed to support smooth interaction and enhance user engagement during rehabilitation sessions. The interface is visually guided, multimodal, and optimized for use on large displays or desktop screens.

### 2.5. Proposed Exercises for Upper Limb Rehabilitation Using Non-Immersive Virtual Reality Using the Ex Unique Short Isometric Daily (EUSIZ) Method

**Figure 7:**
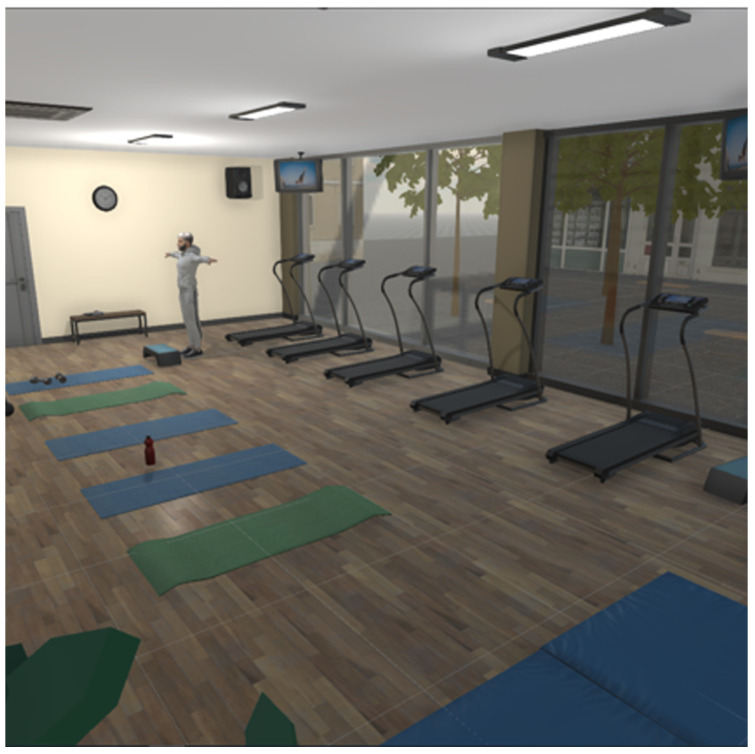
Fitness room.

We recommended beginning the testing session with the first exercise, which is composed of two 3D structures in which three points of interest are positioned for each senior member, the patient’s reference plane being frontal, and the movement of interest pursued was that of abduction, which is a movement of removal of the limb from the median axis of the body. These exercises were developed based on a biomechanical understanding of the upper limb [15]. For the 3D enviroment, a Fitness Room Figure 7 was developend in order to increase user immersion.

The following exercises for upper limb rehabilitation have been designed and analyzed within a specialized physical therapy framework, focusing on patients with acceptable muscle strength. Active muscular mobility is defined as the ability of a muscle to fully mobilize a limb segment against gravity without additional resistance. This level represents the functional threshold for muscle activity, serving as a minimum benchmark for functional capacity.

#### 2.5.1. Muscle Strength

Muscle strength improvement can be achieved through various strategies, particularly isometric contractions, which involve static muscle activation without joint movement. The intensity and duration of these exercises dictate their specific benefits:

- Low-Intensity Contractions (25–35% of Maximum Force): Primarily aimed at maintaining muscle strength, these contractions prevent muscle atrophy.
- Moderate- to High-Intensity Contractions (>35% of Maximum Force): Designed to increase muscle strength, these are generally performed for 3–5 s in patients and up to 12 s in athletes. A single isometric contraction lasting 6 s at high tension can significantly enhance strength, provided that the muscle achieves 60–70% of its maximum force. These range from low-intensity contractions (25–35% of maximum force) for maintaining strength to high-intensity contractions (up to 70% of maximum force) for building strength [16].

In contrast, the ERSIZ technique uses short repetitive isometric contractions of 3–5 s, interspersed with 20 s intervals, performed 20 times daily, ensuring effective recovery while preventing excessive cardiovascular strain.

#### 2.5.2. Restoring Muscle Endurance

Muscle endurance is improved through dynamic exercises that involve low resistance and prolonged activity durations. Weights requiring 15–40% of the maximum force are used, with progressive increases in exercise duration. This approach is particularly effective for enhancing dynamic strength and stamina over time [17].

#### 2.5.3. Gradual Oscilation Technique for Join Mobility

Joint mobility is restored using rhythmic, progressive movements across small to large amplitudes [18]:

- Early-Stage Movements: Small rhythmic amplitudes within the initial arc of motion, focusing on pain reduction.
- Mid-Range Movements: Wider movements without reaching the extremes of the motion arc, ensuring tissue preservation.
- End-Range Movements: Extensive movements reaching the extremes of the arc, respecting tissue resistance.

The analyzed exercises impact the bone and joint systems of the upper limbs, including the shoulders. These components, part of the passive musculoskeletal system, are targeted to improve joint mobility and muscle functionality, as illustrated in Figure 4. The exercises aim to preserve and restore movement amplitudes, enhance circulation, and stimulate tissue trophicity, providing a comprehensive rehabilitation framework. These movements target pain relief and tissue elasticity. VR platforms incorporating these techniques have shown effectiveness in stroke patients [16] and older adults in home settings [17].

## 3. Requirements for Operation

### 3.1. System Requirements

The application requires a PC with Windows 10 or later, equipped with at least an NVIDIA RX 960 graphics card or equivalent, an Intel i3 processor or equivalent, 4 GB of RAM, 1 GB of free disk space, and DirectX 11. Additionally, the Intel RealSense Camera D435i must be connected to the PC via a USB-C port [11]. While the application will function on a standard monitor, we strongly recommend connecting the PC to an LED display, as this allows for a more immersive and visually compelling experience.

### 3.2. Participants and Sample Limitation

Five healthy participants (3 males and 2 females, aged 23–32) with no prior motor impairments were recruited for the study. Participation was entirely voluntary, and all participants provided verbal informed consent prior to taking part. As the study involved no physical or psychological risks, ethical review and approval were waived. The main objective was to evaluate the usability, functionality, and user experience of the non-immersive virtual reality application designed for upper limb rehabilitation. The study focused solely on assessing the system’s usability and user satisfaction, rather than its clinical effectiveness on rehabilitation outcomes.

The small sample size restricts the statistical significance and generalization of the usability findings. Furthermore, it is important to note that none of the participants was actively undergoing rehabilitation. The evaluation focused on assessing the functional operation, real-time feedback, and user interface clarity of the system rather than clinical efficacy. As a result, the current outcomes primarily reflect system performance in a controlled, low-risk environment. Although this preliminary test was essential for validating the core system features, more studies are required to evaluate the effectiveness of the platform in clinical contexts.

## 4. Results

To evaluate the system’s functionality, accuracy, and user satisfaction, a usability test was conducted involving five healthy participants (3 males and 2 females, aged 23–32) with no prior motor impairments. The aim of the test was to assess the usability and user experience of the system, rather than its clinical effectiveness on rehabilitation outcomes. The test was conducted in a laboratory setting using a standard desktop environment with a connected LED display and the Intel RealSense D435i 3D camera positioned 1.5 m from the participant.

### 4.1. System Effectiveness and Usability

To evaluate the system’s effectiveness and perceived usability, a functional prototype was developed and tested with five healthy participants. While the SUS is a general usability instrument, participants were instructed to rate the system specifically in terms of its ease of use, clarity of feedback, and motion interaction within the rehabilitation context. Each participant completed a session using the VR rehabilitation system and was subsequently asked to fill out the System Usability Scale (SUS)—a widely adopted, validated tool used to measure the usability of technological systems through a 10-item questionnaire scored on a 100-point scale. The average SUS score reported in this study was 91.6, indicating a high level of usability. According to the interpretation model proposed by Bangor [18], SUS scores above 68 are considered above average, and scores over 80 are classified as excellent. Therefore, the results place the system within the highest usability tier.

The results, summarized in Table 1, show high levels of movement accuracy (ranging from 89% to 95%) and strong usability ratings, with SUS scores averaging 91.6, indicating excellent usability. These findings suggest that the system is not only effective in tracking and guiding movement but also highly usable and well-received by users, reinforcing its potential as a viable tool in upper limb rehabilitation. These results are consistent with similar VR systems evaluated in stroke recovery settings [15].

### 4.2. Motion Traking System Used

**Figure 8:**
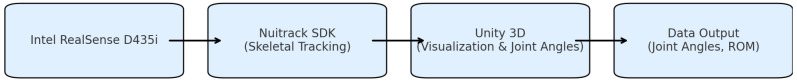
Motion traking system flow.

This study used a custom motion tracking system built with the Nuitrack SDK [16], the Intel RealSense D435i depth camera, and the Unity game engine.Nuitrack is a 3D skeletal tracking middleware that detects and tracks human body joints in real time using a single depth sensor the process flow can be seen in Figure 8. It provides 3D coordinates for key points such as the head, torso, arms, and legs. The Intel RealSense D435i captures depth images, which Nuitrack uses to generate the skeleton data. Unity was used to visualize and record the movement, as well as to calculate joint angles during exercises.

This setup is low-cost and easy to install, making it suitable for rehabilitation, training, and motion analysis in nonlaboratory environments.

### 4.3. Exercise Execution and Movement Accuracy

The participants performed a series of exercises that focused on measuring arm abduction and adduction, which are essential movements in upper limb rehabilitation. Abduction involves lifting the arm away from the body’s midline, while adduction refers to bringing it back toward the body. The system tracked these movements using skeletal detection through the Nuitrack SDK [12]. Each participant completed five repetitions of the abduction–adduction sequence. Movement data were compared against baseline biomechanical models to calculate accuracy. Accuracy was defined as the percentage overlap between expected and actual movement vectors, within an accepted tolerance of ±15 degrees (see Table 2).

### 4.4. Observation and Technical Stability

During testing, no tracking interruptions or system crashes occurred. However, a participant wearing dark clothing experienced brief tracking instability during rotational arm movements. This supports existing recommendations to avoid dark or reflective clothing during motion capture sessions using infrared-based cameras.

### 4.5. Application Performance and Integration of the EUSIZ Method

The proposed non-immersive VR application, based on the Intel RealSense D435i camera and Nuitrack SDK, demonstrated reliable performance in tracking upper limb movements, particularly for gross motor actions such as shoulder flexion/extension and elbow bending. The system performed well during slow and controlled movements, where the visual tracking of joints remained stable and consistent. Real-time visualization in Unity provided clear and immediate feedback, which is especially beneficial in rehabilitation contexts.

However, reduced performance was observed in fine motor tasks or when joints became occluded, such as when the hands were positioned too close to the torso or during fast movements. These limitations stem from the single-camera setup and the natural constraints of depth sensing and joint estimation. To mitigate this, users were instructed to remain within the optimal field of view and maintain clearly visible limb postures throughout the exercises; for this, we created a standing area for the users that keep the system running at the optimal parameters.

The integration of EUSIZ within this framework allows structured daily rehabilitation sessions that are safe, accessible, and repeatable for individuals recovering from stroke or musculoskeletal conditions.

### 4.6. Exercise-Specific Performance Analysis

**Figure 9:**
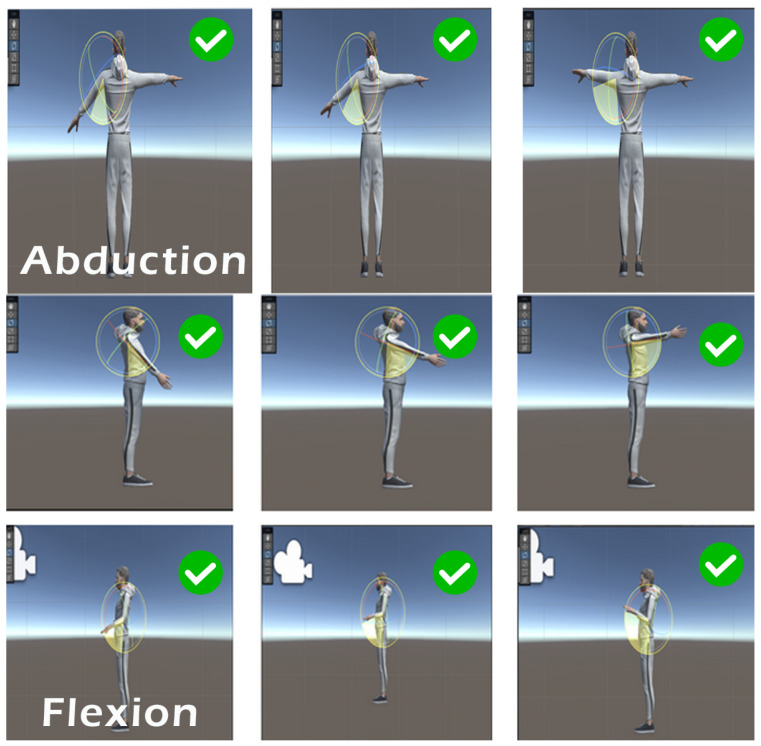
Movements evaluation with a high performance.

**Figure 10:**
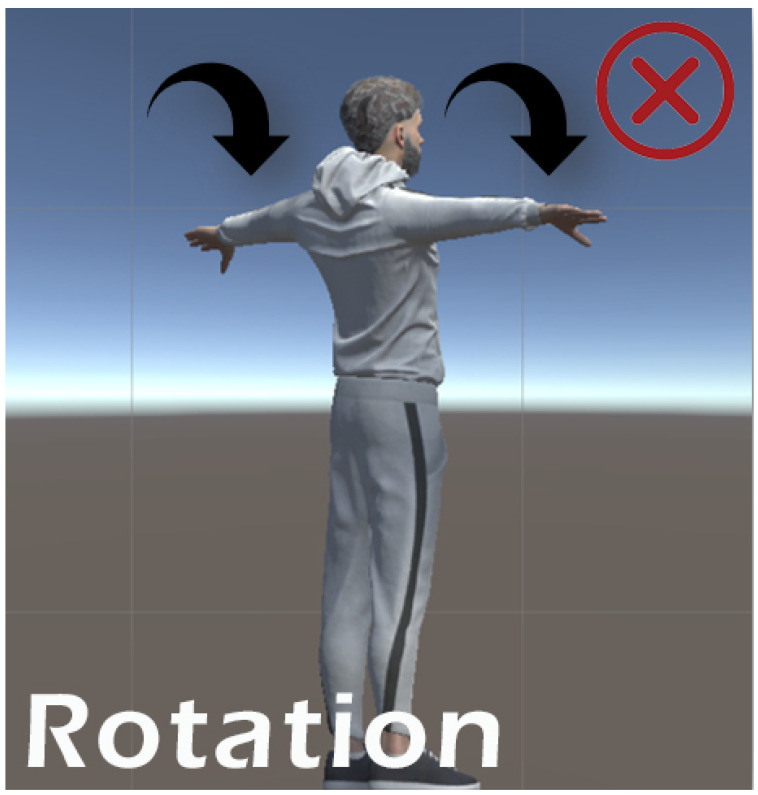
Movements evaluation with a lower perfomance.

Each exercise was evaluated for consistency, sensor accuracy, and participant feedback. Simple, linear movements such as shoulder flexion/extension, elbow flexion, and horizontal abduction/adduction performed very well. These exercises returned high overlap percentages and received the highest user feedback scores. This was largely due to the consistent trajectory and minimal joint complexity, which allowed the RealSense D435i depth camera to accurately track and mirror the movement in the avatar without occlusion. In contrast, exercises such as diagonal reach and arm circles showed decreased performance due to increased movement arc, joint complexity, and instances of the hand exiting the interaction zone. These factors reduced overlap accuracy and led to less stable feedback. Each exercise was evaluated for consistency, sensor accuracy, and participant feedback. The system successfully tracked and mirrored a variety of upper limb movements. Visual examples of the exercises performed in the system including flexion, abduction, and rotational movements are presented in the Figure 9 bellow, where we have marked which movements performed well and which showed reduced performance Figure 10.

### 4.7. System Perfomance Indicatior and Implementation Obstacles

Key performance indicators included the System Usability Scale (SUS) score, overlap percentage (computed internally through joint-to-target trajectory comparisons), visual feedback latency, and motion fluidity. Obstacles encountered during development and testing included the following:

- sensor misalignment after repeated use;
- calibration drift in RealSense tracking requiring periodic resets;
- depth camera occlusion during fast or complex joint motion;
- minor UI lag during multi-joint movements.

Most of these were mitigated through improved software-side smoothing, better calibration protocols, and UI interaction zone visual aids.

### 4.8. Evaluation of the EUSIZ Method

The EUSIZ method was highly effective in exercises with confined movement arcs and consistent postures, such as shoulder flexion and elbow flexion. It successfully tracked movement within the pre-defined interaction zone and returned stable visual feedback. However, EUSIZ struggled to capture dynamic actions like arm circles or diagonal reaches, where limbs temporarily exited the capture area or occluded other joints. These exercises resulted in inconsistent feedback quality and lower performance ratings.

#### Exercise Performance Chart

The Table 3 helps determine which exercises are best suited for inclusion in the expanded version of the application and highlights areas that may require further optimization.

### 4.9. Participation Feedback

Participants were asked to describe their overall experience, perceived ease of use, clarity of the visual feedback, and comfort during the exercises. Most users reported that the system was intuitive and easy to follow, and appreciated the visual representation of their movements in real time. Some participants suggested improving joint tracking stability during fast movements and adding sound cues to enhance guidance.

### 4.10. Potential Real-Life Implication

The proposed non-immersive VR system offers a practical and cost-effective solution for upper limb rehabilitation in home or outpatient settings. By providing real-time visual feedback and supporting structured routines like the EUSIZ method, the system encourages regular physical activity, improves engagement, and supports remote monitoring by therapists. This approach has the potential to reduce rehabilitation costs, improve therapy adherence, and extend access to quality care beyond clinical environments.

## 5. Discussion

This study explores the potential of non-immersive virtual reality (VR) architecture using a 3D vision camera system to aid in upper limb rehabilitation. Our findings indicate that this system effectively tracks arm movements such as flexion and abduction, utilizing the EUSIZ method to enhance muscle strength, restore range of motion (ROM), and rebuild muscle memory. The user feedback from the prototype testing suggests high levels of usability and satisfaction, indicating that non-immersive VR systems can be intuitive and accessible for rehabilitation purposes.

The findings align with previous research demonstrating the benefits of VR in rehabilitation settings. In a randomized controlled pilot trial reported that immersive VR-based cognitive training significantly improved both functional abilities and patient engagement in individuals with chronic stroke [19] also other studies have shown that VR applications improve psychological and functional outcomes by engaging patients in gamified environments that encourage active participation and enhance motivation [20]. Specific VR rehabilitation protocols have been shown to outperform generalized ones [21], and even people suffering from stroke benefit from virtual walking platforms [22]. RealSense camera-based systems have also demonstrated utility in tracking motion accuracy and user outcomes in rehabilitation contexts [23]. Virtual simulations of daily activities (ADLs) also offer valuable tools for upper limb motor recovery [24]. Frameworks for home upper limb telerehabilitation using VR and wearable sensing technologies have also shown promising results [25]. Recent meta-analyses further confirm the efficacy of VR-based interventions for improving upper limb function in post-stroke patients [26] and for neurologic disorders [27]. Non-immersive VR systems have also been shown to be particularly effective in neurological rehabilitation, offering accessible and user-friendly solutions for patients with varying impairments [21].

The integration of the EUSIZ method provides a structured approach to improving muscle strength and joint mobility, complementing the findings that isometric contractions are beneficial to maintaining and improving muscle functionality. Despite the promising results, several limitations must be acknowledged. First, a sample size of five healthy participants is small, and the results may not fully generalize to patients with varying levels of impairment or different rehabilitation needs. Second, the non-immersive nature of the system, while accessible, may lack the full sensory engagement of immersive VR systems, potentially limiting its effectiveness in certain therapeutic contexts. Furthermore, reliance on specific hardware, such as the Intel RealSense D435i camera, may pose compatibility challenges for widespread adoption.

### 5.1. Future Research and Applications

In the future, the system may incorporate machine learning for adaptive personalization [20] and AI-assisted rehabilitation programs. In addition, machine learning algorithms could be explored to optimize exercise recommendations based on individual progress. Comparative studies between immersive and non-immersive systems will also be conducted to identify the most suitable applications for each approach [28]. Previous randomized controlled trials have highlighted significant differences in rehabilitation outcomes between immersive and non-immersive VR modalities [28]. Furthermore, systematic reviews have reinforced the effectiveness of VR-based rehabilitation programs in enhancing upper limb motor function and daily living activities in post-stroke populations [2].

### 5.2. Study Limitation

A key limitation of this study is that all participants were healthy individuals without motor impairments. Although this allowed us to evaluate system stability, it does not fully reflect the challenges faced by clinical populations. Future studies are needed to test the system in real rehabilitation contexts, where there are variations in movement patterns.

## 6. Conclusions

A functional prototype has been developed to track and improve upper limb, incorporating a structured exercise protocol. Using a non-immersive virtual reality system, this approach enables the precise measurement of movements while offering a comprehensive perspective on user progress and rehabilitation outcomes. Virtual reality emerges as a transformative technology in rehabilitation engineering. With its ability to analyze body movements and create engaging, interactive environments, VR not only fosters physical recovery but also addresses neurological aspects, paving the way for innovative therapeutic applications and broader possibilities in healthcare.

Virtual reality is not universally suitable and must be tailored to meet the specific needs of patients. As an emerging technology, VR continues to evolve, with advancements in hardware and ongoing research shaping its capabilities. These developments are critical for maximizing its potential and ensuring its effectiveness in diverse rehabilitation contexts. Building on this foundation, our future research will focus on integrating immersive virtual reality using head-mounted displays (HMDs) to enhance engagement, depth perception, and spatial awareness during rehabilitation exercises. This aligns with recommendations from previous studies using similar technologies [29]. Full immersion offers several advantages, including increased presence, greater motivation, and more natural interaction with virtual environments, which can improve motor learning and adherence to therapy. By incorporating these elements, we aim to create a more effective and engaging rehabilitation experience. Additionally, we will continue to develop and refine our system, leveraging advancements in VR and motion tracking to provide more personalized rehabilitation solutions. This ongoing research will contribute to expanding VR’s role in therapeutic applications, ensuring its adaptability and effectiveness across a wide range of rehabilitation needs. As VR technology continues to evolve, its applications in rehabilitation will expand, contributing to more effective, scalable, and accessible therapeutic solutions for individuals recovering from upper limb impairments.

## Figures and Tables

**Table 1:** User movement accuracy and usability evaluation based on the System Usability Scale (SUS).

User	Accuracy of the Movement (%)	SUS Score
User 1	94%	93
User 2	91%	90
User 3	95%	95
User 4	92%	91
User 5	89%	89

SUS: System Usability Scale; based on user perception of interface usability and feedback.

**Table 2:** Movement accuracy and performance analysis for arm abduction and adduction exercises.

Participant	Avg. Movement Accuracy (%)	Most Accurate Movement	Least Accurate Movement
User 1	94%	Abduction	Adduction
User 2	91%	Adduction	Abduction
User 3	95%	Abduction	Adduction
User 4	92%	Adduction	Abduction
User 5	89%	Adduction	Abduction

Data based on skeletal tracking during arm abduction and adduction exercises.

**Table 3:** Performance evaluation of rehabilitation exercises.

Exercise	Tracking Accuracy	Overlap (%)	User Feedback (1–5)	Stability in EUSIZ Zone
Shoulder Flexion/Extension	High	92	5.0	Stable
Horizontal Abd/Adduction	High	88	4.8	Stable
Elbow Flexion	High	90	4.9	Stable
Arm Circles	Medium	76	3.7	Partial
Lateral Raise	Medium	79	3.9	Partial

## Data Availability

All data generated or analyzed during this study are included in this published article. No additional datasets are available.

## Abbreviations

The following abbreviations are used in this manuscript:

VR	Virtual Reality
SDK	Software development kit
SW	Software
HW	Hardware
EUSIZ	Ex Unique Short Isometric Daily
SUS	System Usability Scale
RGB	Red, Green, Blue
